# Life Course Neighbourhood Deprivation and Self-Rated Health: Does It Matter Where You Lived in Adolescence and Do Neighbourhood Effects Build Up over Life?

**DOI:** 10.3390/ijerph181910311

**Published:** 2021-09-30

**Authors:** Stephen Jivraj, Owen Nicholas, Emily T. Murray, Paul Norman

**Affiliations:** 1 Institute of Epidemiology and Health Care, University College London, London WC1E 6BT, UK; Emily.murray@ucl.ac.uk; 2 Department of Statistical Science, University College London, London WC1E 6BT, UK; o.nicholas@ucl.ac.uk; 3 School of Geography, University of Leeds, Leeds LS2 9JT, UK; p.d.norman@leeds.ac.uk

**Keywords:** neighbourhood effects, neighbourhood deprivation, Townsend index, cumulative effects, multilevel modelling, self-rated health, life course epidemiology

## Abstract

There is an overreliance on concurrent neighbourhood deprivation as a determinant of health. Only a small section of the literature focuses on the cumulative exposure of neighbourhood deprivation over the life course. This paper uses data from the 1958 National Child Development Study, a British birth cohort study, linked to 1971–2011 Census data at the neighbourhood level to longitudinally model self-rated health between ages 23 and 55 by Townsend deprivation score between ages 16 and 55. Change in self-rated health is analysed using ordinal multilevel models to test the strength of association with neighbourhood deprivation at age 16, concurrently and cumulatively. The results show that greater neighbourhood deprivation at age 16 predicts worsening self-rated health between ages 33 and 50. The association with concurrent neighbourhood deprivation is shown to be stronger compared with the measurement at age 16 when both are adjusted in the model. The concurrent association with change in self-rated health is explained by cumulative neighbourhood deprivation. These findings suggest that neglecting exposure to neighbourhood deprivation over the life course will underestimate the neighbourhood effect. They also have potential implications for public policy suggesting that neighbourhood socioeconomic equality may bring about better population health.

## 1. Introduction

This paper aims to overcome a common limitation in neighbourhood effects research which is a reliance on concurrent neighbourhood of residence to determine whether there is anything about the places individuals live in which impacts on their health [1]. There is a growing evidence-base suggesting it is not simply where you live today, rather your cumulative neighbourhood history; considering duration of exposure to a neighbourhood and the impact of both in situ neighbourhood change and migration between neighbourhoods. It is important to take account of neighbourhood histories because people may remain connected to neighbourhoods they have previously lived in by way of at least two of the processes identified by Galster [2] in a seminal paper on how neighbourhood can causally affect individual outcomes: through (1) continued social ties with family and friends and (2) institutional ties with schools and other services in a previous neighbourhood [3]. Here, we focus on neighbourhood socioeconomic deprivation often measured through a composite of indicators (e.g., unemployment, overcrowding, household tenure and education) or a single item (e.g., income or unemployment) and provide a summary of the evidence on how exposure to more deprived neighbourhoods over time impacts on health.

Research on life course neighbourhood effects remains rare because of the lack of historical geocoded information available in nationally representative longitudinal studies or that longitudinal studies are simply not mature enough to analyse the relationship between neighbourhood deprivation and health over a long enough period [4,5]. Yang and South [6] derived four trajectories of exposure to high poverty neighbourhoods using the US National Longitudinal Survey of Youth, 1979 Cohort, to compare the strength of association between these trajectories with self-rated health at around age 40 against the association with neighbourhood poverty in 1980 (when respondents were in their early twenties) and neighbourhood poverty in 2010 (at the same time they were asked about their health). They found that neighbourhood poverty trajectories were a stronger predictor of midlife health than the point in time neighbourhood poverty measure, leading them to conclude that a life-course approach is important to understand the extent of neighbourhood effects. Clarke et al.’s [7] work supports these findings using a different US cohort study, Americans’ Changing Lives, though the duration of exposure analysed was shorter at 15 years and there was no direct comparison to concurrent measures of neighbourhood deprivation. The outcome in the Clarke et al. study was functional decline in adults aged 25 and over.

There is also support for cumulative exposure to neighbourhood deprivation being associated with health using longitudinal data from Scandinavia. Gustafsson [8] found that functional somatic symptoms at age 42 in women were associated with cumulative neighbourhood deprivation from age 16 using the Northern Swedish Cohort of school leavers in the municipality of Luleå. The same conclusion is drawn from the same dataset on the relationship between allostatic load using 12 biological markers and cumulative neighbourhood deprivation [9]. As well, Prior [10], in this Special Issue, found that continued exposure to neighbourhood deprivation over a 20-year period was associated with higher allostatic load using data from the British Household Panel Study. She used an adult sample (mean age 52) to create four neighbourhood deprivation trajectories using latent class growth analysis. Kivimäki et al. [11] also found that people who were consistently exposed to high neighbourhood deprivation in Finland were more likely to be hypertensive and have diabetes, both measured objectively, using data from the Young Finns Study of children aged 6–18 years in 1980 or 1983 and who were followed up over a 31 year period.

Returning to studies that test for the strength of association between concurrent and cumulative neighbourhood deprivation and health in the same model, Ellaway et al. [12] measured telomere length, which has been suggested to indicate biological ageing, and its association with individual perceptions of neighbourhood quality over a 20 year period. They used data from the Scotland Twenty-07 study which started in 1987 with individuals aged 15, 35 and 55. The individuals were asked about the neighbourhood quality in 1995, 2000 and 2007. Telomere length was measured from blood analytes in 2007. They found shorter telomere length in women who consistently reported their neighbourhood quality as poorer. The association was only significant in 2007 when comparing concurrent measures of poor neighbourhood quality separately. Hagedoor and Helbich [3] and Pearson et al. [13] also suggest concurrent neighbourhood is a stronger determinant of health when also taking into account a cumulative measure of neighbourhood deprivation. Hagedoor and Helbich used Dutch register data on neighbourhood histories of people aged 40–64 and their unemployment rate over 15 years, and found those living in 300 metre square buffers with higher unemployment are less likely to commit suicide compared with those living in neighbourhoods with lower unemployment. Pearson et al.’s ecological analysis of cardiovascular mortality over 15 years used data from New Zealand and found that current neighbourhood deprivation is a stronger predictor compared with a classification of a neighbourhood deprivation trend. 

The outcome of choice may explain the difference in findings on whether concurrent or cumulative exposure to neighbourhood deprivation is the stronger determinant of health. It is also the case that studies suggesting concurrent neighbourhood deprivation is more important than cumulative neighbourhood deprivation have measured neighbourhood deprivation from an adult baseline age. It might be the case that most, if not all, of a neighbourhood effect has already taken hold by this stage in the life course. For the rest of a person’s life there could be only a marginal, if any, additional cumulative effect. It is therefore important to measure neighbourhood deprivation in childhood to determine a fuller effect. 

We aim to overcome the reliance on concurrent neighbourhood deprivation to test neighbourhood effects using a family of longitudinal models and ask three research questions under the assumptions of these models: (1) is neighbourhood deprivation at age 16 related to self-rated health up to age 55; (2) if there is, then is the relationship between neighbourhood deprivation at age 16 and change in self-rated health up to age 55 explained by neighbourhood deprivation at intermediate ages; and (3) are changes in self-rated health up to age 55 explained by variation in cumulative values of neighbourhood deprivation up to that age, or concurrent values at that age? We use Census data, 1971–2011, linked to a 1958 British birth cohort study, which enables us to build on the existing evidence base by using a long period of follow up from childhood and measuring change in health up to midlife in a nationally representative sample. 

## 2. Materials and Methods

### 2.1. Data

**Figure 1:**
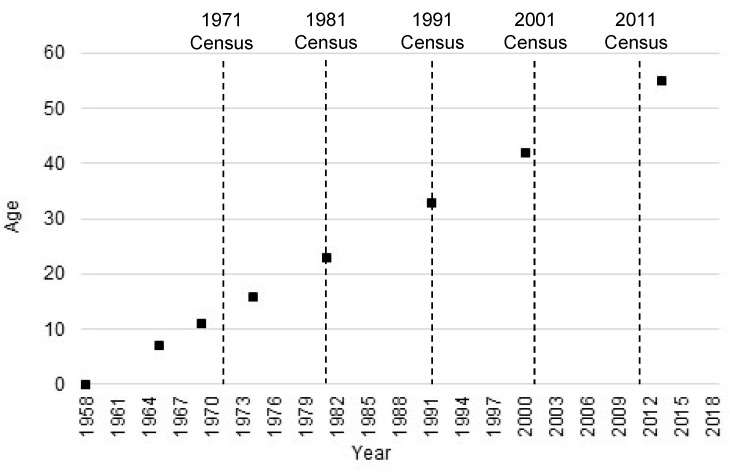
1958 National Child Development Study age and year plan.

Individual data are taken from the 1958 National Child Development Study (NCDS), which is an interdisciplinary study of births in England, Scotland and Wales in a single week of 1958 who have been followed up at various time points ever since [14]. In this paper we use data collected through face-to-face interviews and telephone interviews at ages 7, 11, 16, 23, 33, 42, 50 and 55. The place of residence is matched to census data from age 16 onwards using 2011 lower super output areas (LSOAs) in England and Wales and data zones in Scotland [15]. Both, which will be referred to as LSOAs hereafter, are spatial containers created from the outputs of British census data to have even population size. They have been used routinely as a proxy for neighbourhoods in health geography although they do not attempt to represent communities as perceived by the people who live in them [16]. It is not possible to match survey respondents to LSOAs before age 16 because address information is incomplete. Figure 1 shows the age at survey against year of survey with the census dates marked.

### 2.2. Measures

The outcome variable is self-rated health, a measurement generally perceived to indicate general health and shown to be strongly associated with mortality and morbidity in adults [16,17]. In this study, self-rated health is derived from a question that asks how is your health in general? The question was first asked in NCDS at age 23 (1981) when the response categories were excellent, good, fair or poor, and again asked in the same way at ages 33 (1991) and 42 (2000). An additional response category of very good was used at ages 50 (2008) and 55 (2013). To ensure comparability between ages 23 and 55 a three-category (good, fair and poor) self-rated health variable was harmonised across survey years. 

The exposure variable is the Townsend deprivation score at ages 16, 23, 33, 42, 50 and 55 at the LSOA level. The Townsend deprivation score is a standardised (mean = 0, standard deviation = 1) summary score of four census measured variables: household overcrowding, unemployment, non-home and non-car ownership, with a higher value indicating greater deprivation [18]. The measure is widely used in health research to indicate area deprivation [1,19]. The Townsend scores were derived by apportioning values at each decennial census, 1971–2011, to 2011 LSOA boundaries. Values for NCDS survey years were calculated using linear interpolation between censuses [20]. Cumulative Townsend deprivation scores were calculated by summing values up to and including each survey year.

A variable was added to the statistical models (see below) for year of survey (1981, 1991, 2000, 2008 and 2013) as well as non-time varying individual characteristics (sex at birth and childhood poverty, health and social class) and time varying individual characteristics (social class and marital status). These may explain selection into neighbourhoods across the life course as well as variation in self-rated health and are therefore measured at first occurrence at age 16 or before. Childhood poverty was measured by whether a respondent claimed free school meals at age 11. Childhood health was measured by whether a respondent had missed more than a month of school due to ill health at age 11. Childhood class was measured using Registrar General’s Social Class of a respondent’s father at age 16 and split into professional, non-manual skilled, manual skilled and semi or unskilled. Values were taken at age 11 or 7 if they were missing at older childhood ages. Social class at each adult survey was measured using the Registrar General’s Social Class of the respondent’s occupation at the time of survey. Marital status was taken at each adult survey, dichotomised into married and not married (i.e., single and never married, separated, divorced or widowed). 

### 2.3. Statistical Analysis

The three-category self-rated health outcome was analysed using multilevel ordinal models where survey year at level 1 is nested within an individual respondent at level 2. A three-level model was fitted to take into account between LSOA variation. When variance at the LSOA level was estimated it was very small as a proportion of the total variance across all levels and not statistically significant; therefore, two-level models are presented here. Models were fitted in a series of steps where in each model the outcome Y_tj_ is self-rated health at survey year t for respondent j. Y_tj_ is modelled by ordinal logistic regression with a latent variable whose value is a random coefficient for respondent j plus a linear combination of non-time and time varying variables. The random coefficients were modelled as normally distributed. Models including random slopes on survey year (i.e., testing whether the self-rated health change over time varies between respondents) would not converge. We used the *meologit* command in Stata 16.0. Random effects models are commonly used, for example Kivimäki et al. [11], but are difficult to interpret causally because they neglect plausible pathways of influence.

The first model responds to the first research question set out above as to whether neighbourhood deprivation at age 16 predicts change in self-rated health up to age 55; hence, it was fitted to include the Townsend deprivation at age 16 as well as non-time varying controls (sex, child poverty status, child health and child class) and time-varying controls (adult class and marital status). The second model aims to answer the second research question on whether concurrent neighbourhood deprivation is a more important predictor of self-rated health compared with a measure at age 16. Hence, model two includes concurrent Townsend deprivation at the survey at which the outcome is measured. The final model provides a response to the third research question on whether concurrent or cumulative neighbourhood deprivation up to age 55 explains more of the variation in self-rated health at the same age. This final model then adds the cumulative Townsend score to model 2, itself calculated by taking the summed score up to the wave in which the outcome was measured. All models include interactions between survey year and Townsend deprivation score, whether at age 16, concurrent year or cumulatively, to determine trajectories of self-rated health by neighbourhood deprivation. 

A complete case analysis would have reduced the original NCDS sample by around two-thirds. The main source of the missing data was cumulative Townsend scores, which requires a respondent to be present at each survey and that their address matches to a 2011 LSOA (see Table 1). There was missingness in the region of 30–40% due to missing values across survey years for the adult social class, marital status, self-rated health and Townsend score. To minimise the non-response bias and to increase the analytical sample size, multiple imputation by chained equations (25 imputed datasets) was used to impute values across survey years for respondents who had not died or emigrated. 

## 3. Results

Table 1 shows the sample characteristics by survey year for all variables used in the models. The proportion of respondents reporting fair or poor self-rated health increased from ages 23 to 55 (1981 to 2013). For example, less than 1% reported poor health at age 23 compared with 6% at age 55. The mean concurrent Townsend deprivation score was lower in later survey years, which means respondents in 2013 were living in LSOAs that were relatively less deprived on the national scale compared with respondents in 1981. Respondents were living in LSOAs, on average, 0.35 standard deviations above the national mean in 1981, whereas in 1991 and later survey years they were living, on average, in LSOAs below the national mean. The mean for respondents in 2013 was more than 1 standard deviation below the national mean. This may reflect three processes: relative improvements in the LSOAs NCDS respondents were living in, net moves to relatively less deprived LSOAs or selective attrition. There is some evidence for the latter from the lower Townsend scores at age 16, lower individual poverty rate and higher child social class in respondents in 2013 compared with earlier survey years. This is because these are non-time varying characteristics that should remain constant over time unless the study sample selectively reduces in size. There is also evidence for either improvements in the LSOAs NCDS respondents were living or net movements to less deprived LSOAs because the mean for the concurrent LSOA Townsend score (−1.03) is above the cumulative Townsend score (−0.79), suggesting respondents have lived in more deprived LSOAs at earlier points in their lifetime. During a similar time period, ONS Longitudinal Study (LS) members had equivalent area deprivation experiences [21]. Disentangling the relative contribution of in situ change and internal migration is outside the bounds of this paper, though the ONS LS and other data indicate both processes are influential, the latter more than the former [22,23]. 

Table 2 presents the multilevel ordinal model results with each model providing a response to our three research questions. Model 1 includes year of survey, Townsend deprivation scores for the LSOA of residence at age 16, an interaction between year of survey and Townsend score at age 16 and control variables. Model 2 adds concurrent Townsend deprivation score and an interaction with survey year. Townsend score at age 16 is removed and cumulative Townsend score is added along with an interaction with survey year in Model 3.

Model 1 shows the log odds of reporting a category of self-rated health worse than good (i.e., fair or poor) for a unit increase in Townsend score (i.e., more deprived) at age 16 is 0.04 (SE 0.02), with a 0.05 larger difference for respondents in both of the years 2000 (SE 0.02) and 2008 (SE 0.02) compared with respondents in 1981. There was no significant difference for respondents in 1991 and 2013 relative to 1981. This suggests that those living in more deprived LSOAs at age 16 report poorer health as they age from 33 to 50. Model 2 shows that when concurrent Townsend score is added to model 1, the association between Townsend score at age 16 and self-rated health is attenuated. The association of concurrent self-rated health with Townsend score is robust to adjustment for Townsend score age 16, with the association greater for respondents in the 2000, 2008 and 2013 surveys compared with respondents in 1981. Model 3 shows that the concurrent Townsend score is not significantly related to change in self-rated health over survey year when taking into account the cumulative Townsend score. A unit increase in the cumulative Townsend score for respondents in 1981 is not significant, but then this association strengthens with almost every survey year. The 2013 cumulative Townsend score is associated with a 0.15 (SE 0.05) log odds greater increase in reporting fair or poor self-rated health compared with cumulative Townsend score in 1981. This suggests neighbourhood deprivation experienced between 16 and 55 builds up a disadvantage that is associated with worse self-rated health over time. 

**Figure 2:**
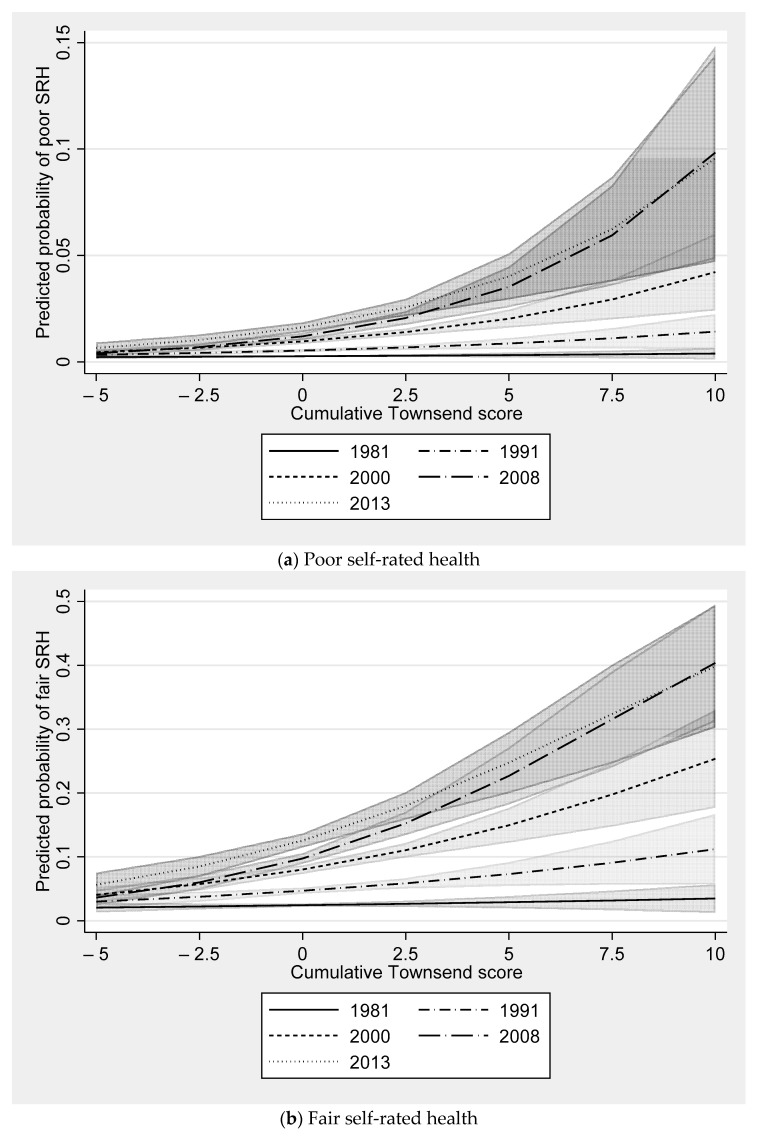

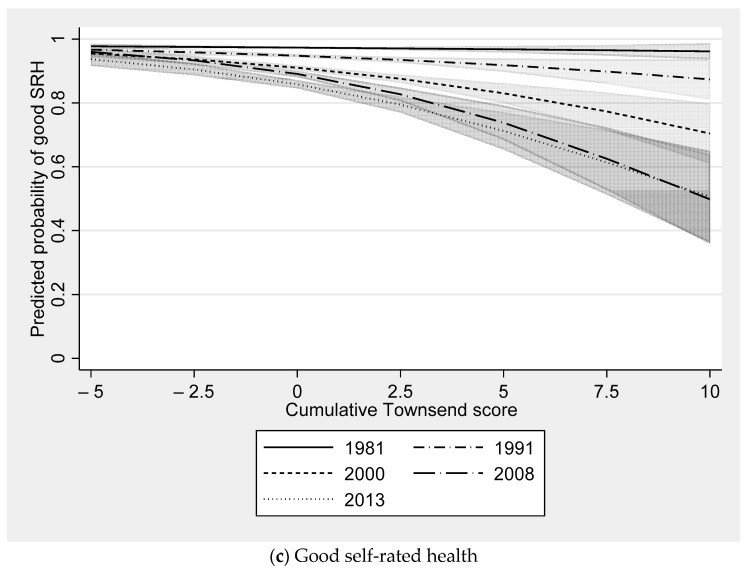
Predicted probability of self-rated health by survey year and cumulative Townsend score. Notes: shaded area around each line represents 95% confidence intervals.

Figure 2 shows how the predicted probability of the three self-rated health categories (good, fair and poor) for each survey year varies over cumulative Townsend score. There was a considerably lower probability of good self-rated health at progressively later survey years as the cumulative Townsend score is higher (see Figure 2a). For example, in 1981 there was very little difference in the predicted probability of good self-rated health at higher levels of deprivation. At a cumulative Townsend score of −3, which is approximately the 10th percentile (i.e., a relatively less deprived neighbourhood), and a cumulative Townsend score of 3, which is approximately the 90th percentile (i.e., a relatively more deprived neighbourhood), the predicted probability is near identical (differing by less than 0.01). The same gap in the predicted probability of good self-rated health was 0.05 by 2000 and more than 0.15 in 2008 and 2013. The relative difference between the same points on the cumulative Townsend distribution across survey years was similar for the predicted probability of fair and poor self-rated health (see Figure 2b,c).

## 4. Discussion

There is a plethora of studies in the field of health geography suggesting your health is affected by the neighbourhood context you live in. Most of this evidence base relies on a measurement of contemporaneous neighbourhood deprivation that assumes the exposure is evenly felt by those who have spent their entire life in their current neighbourhood, those who are recent in-movers and those who have seen the neighbourhoods they have lived in deteriorate or improve socioeconomically. We find the level of neighbourhood deprivation at age 16 predicts change in self-rated health from age 33 to 50. This is largely explained by concurrent neighbourhood deprivation to the time self-rated health was measured. The association between concurrent neighbourhood deprivation and change in self-rated health is itself fully attenuated by the inclusion of a cumulative measure of neighbourhood deprivation from age 16 to 55. This finding supports the balance of existing research suggesting cumulative exposure to neighbourhood deprivation is an important determinant of health [6,7,8,9,10] and more important than concurrent neighbourhood deprivation when directly tested [6,24].

What is remarkable about this body of evidence that broadly comes to the same conclusion is that measures of cumulative neighbourhood deprivation have been operationalised using a variety of methods, conceptually different indicators, a range of spatial scales considered to represent neighbourhoods and in different national contexts [4]. Yang and South [6] group neighbourhood poverty rates for US census tracts using latent class analysis; Prior [10] groups LSOAs by Townsend deprivation scores also using latent class analysis; Kivimaki et al. [11] group 250 m grid squares by a composite measure of unemployment, social renting and education by severity; Clarke et al. [7] use principal component factor scores for US census tracts from nine indicators; Gustafsson [8,9] use summed scores for Swedish small area market statistics areas from eight indicators. The studies which suggest concurrent neighbourhood deprivation is a stronger determinant of health have not measured residential history before adulthood and measure cause-specific mortality of working age populations [3,13], both of which make it difficult to determine a life course effect of neighbourhood deprivation on health.

Our findings confirm the suggestion by Yang and South [6] that the association between earlier life or concurrent neighbourhood deprivation and health is likely to be capturing the influence of life course exposure to neighbourhood deprivation. This is perhaps because, as Murray et al. [25] in this Special Issue suggest, neighbourhood deprivation is tracked across the life course (i.e., if you lived in a poor neighbourhood in childhood, you continue to live in a poor neighbourhood for the rest of your adult life even if you move between neighbourhoods). It is therefore not essential to measure cumulative neighbourhood deprivation across the life course to estimate an association between neighbourhood deprivation and health. Nonetheless, there might well be an underestimation of the relationship without it.

The implication of these findings points to a requirement to improve neighbourhood deprivation for individuals at as early an age as possible to have the greatest impact on improving population health. Governments, local and national, in diverse geopolitical contexts have instigated area-based interventions to improve the places individuals live in with the aim of improving life outcomes. These findings support a continuation of these policies. In the UK, area-based policies have been enacted in various forms since the 1950s [26] yet the persistence in inequality between rich and poor places remains remarkably constant [27]. There is some suggestion that the interventions of the Labour government (1997–2010) through a plethora of neighbourhood-based interventions reduced health inequalities, including New Deal for Communities [28,29] and Sure Start [30]. The retrenchment in these programmes after 2010 has been shown to be associated with widening inequalities between deprived areas and less deprived areas [31,32], suggesting a renaissance of area-based interventions could help to meet the current governments aim to tackle health inequalities as set out in the strategic aims of the newly formed Office for Health Improvement and Disparities [33].

The next question ought to be: is this causal? There is a suggestion that it is not selective migration, at least not after childhood, and therefore what is it about neighbourhoods that apparently makes people unhealthier? To address this, two advancements are required. First, methods of causal analysis to test the neighbourhood effect hypothesis more convincingly. Whether this is through study designs that lend themselves to causal inference, such as randomised experiments, or methods including observational quantitative data analysis or qualitative analysis, that with reasoned assumptions can allow causal claims. Second, more research is required on the specific processes through which a neighbourhood can impact on an individual’s life. Is it that deprived neighbourhoods harbour the transmission of poor health information that is negatively acted upon by many residents and other socialisation processes, or is it the institutional, environmental or geographical processes identified by Galster [2]?

## 5. Conclusions

In summary, it is important to consider life course neighbourhood deprivation to determine the fullness of its effect. Additionally, if we were to allow ourselves to interpret our findings causally, improving neighbourhood deprivation should have a greater effect on population health if achieved and maintained as early in life as possible.

## Figures and Tables

**Table 1:** Sample characteristics by survey year, National Child Development Study, 1981–2013.

Mean	1981 (*n* = 16,402)		1991 (*n* = 16,174)		2000 (*n* = 16,091)		2008 (*n* = 15,806)		2013 (*n* = 15,613)		Total (*n* = 80,086)	
	Mean (SD) or % ^1^	Mis. (%) ^2^	Mean (SD) or % ^1^	Mis. (%) ^2^	Mean (SD) or % ^1^	Mis. (%) ^2^	Mean (SD) or % ^1^	Mis. (%) ^2^	Mean (SD) or % ^1^	Mis. (%) ^2^	Mean (SD) or % ^1^	Mis. (%) ^2^
Self-rated health		23.6%		30.3%		29.3%		38.4%		42.1%		32.6%
Good	90.4%		86.4%		81.8%		81.5%		80.0%		84.4%	
Fair	8.7%		11.9%		14.7%		12.7%		14.0%		12.2%	
Poor	0.9%		1.8%		3.6%		5.7%		6.0%		3.4%	
Townsend deprivation score												
Age 16	0.30 (3.00)	26.6%	0.30 (3.00)	27.1%	0.30 (3.00)	27.1%	0.29 (3.00)	27.0%	0.28 (3.00)	27.1%	0.29 (3.00)	27.0%
Concurrent	0.35 (3.30)	25.2%	−0.42 (2.95)	30.4%	−0.68 (2.46)	29.8%	−0.78 (2.46)	28.3%	−1.03 (2.48)	43.0%	−0.48 (2.8)	31.3%
Cumulative	0.34 (2.77)	34.0%	−0.06 (2.44)	48.0%	−0.31 (2.21)	54.2%	−0.66 (1.98)	62.8%	−0.79 (1.93)	66.9%	−0.19 (2.41)	53.0%
Sex at birth		0.0%		0.0%		0.0%		0.0%		0.0%		0.0%
Female	48.7%		48.8%		48.9%		49.1%		49.3%		49.0%	
Childhood poverty		18.5%		18.4%		18.5%		18.5%		18.5%		18.5%
Yes	10.2%		10.3%		10.2%		10.1%		10.0%		10.2%	
Childhood ill health		19.3%		19.4%		19.3%		19.3%		19.3%		19.3%
Yes	5.7%		5.8%		5.7%		5.7%		5.7%		5.7%	
Childhood social class		11.2%		11.2%		11.2%		11.2%		11.2%		11.2%
Professional	22.4%		22.3%		22.3%		22.4%		22.6%		22.4%	
Non-manual skilled	11.4%		11.4%		11.4%		11.4%		11.4%		11.4%	
Manual skilled	41.7%		41.7%		41.7%		41.8%		41.7%		41.7%	
Semi or unskilled	24.5%		24.5%		24.5%		24.4%		24.2%		24.4%	
Adult social class		39.5%		34.6%		40.4%		47.9%		54.6%		43.4%
Professional	19.3%		36.2%		42.9%		47.6%		48.6%		37.9%	
Non-manual skilled	34.0%		23.6%		21.3%		19.9%		19.3%		24.1%	
Manual skilled	24.7%		20.3%		20.2%		19.0%		18.2%		20.7%	
Semi or unskilled	22.0%		20.0%		15.6%		13.6%		13.8%		17.4%	
Marital status		23.6%		32.0%		29.4%		38.1%		41.5%		32.8%
Married	44.6%		70.6%		70.8%		68.8%		70.3%		64.2%	

^1^ Sample characteristics of valid respondents (i.e., non-missing sample); ^2^ percent missing respondents of total survey year sample size.

**Table 2:** Multilevel ordinal logistic regression estimates predicting worse than good self-rated health.

	Model 1			Model 2			Model 3		
Fixed effects	Coef.	SE		Coef	SE		Coef	SE	
Townsend score									
Age 16	0.041	0.016	*	0.023	0.018				
Concurrent				0.033	0.016	*	0.046	0.029	
Cumulative							0.036	0.035	
Survey year (ref. 1981)									
1991	0.720	0.055	***	0.709	0.054	***	0.705	0.054	***
2000	1.287	0.054	***	1.299	0.054	***	1.295	0.054	***
2008	1.513	0.058	***	1.551	0.058	***	1.527	0.059	***
2013	1.804	0.064	***	1.847	0.065	***	1.833	0.066	***
Survey Year * Age 16									
1991	0.016	0.017		0.019	0.021				
2000	0.055	0.017	**	0.044	0.020	*			
2008	0.047	0.018	*	0.026	0.021				
2013	0.025	0.018		0.010	0.020				
Survey Year * Concurrent									
1991				0.010	0.021		−0.021	0.038	
2000				0.068	0.020	**	0.003	0.036	
2008				0.116	0.022	***	−0.020	0.038	
2013				0.100	0.020	***	−0.017	0.039	
Survey Year * Cumulative									
1991							0.061	0.043	
2000							0.116	0.041	**
2008							0.185	0.045	***
2013							0.151	0.046	**
Sex at birth (ref. male)									
Female	0.130	0.051	*	0.126	0.050	*	0.126	0.050	*
Child poverty (ref. no)									
Yes	0.798	0.084	***	0.733	0.083	***	0.676	0.082	***
Child ill health (ref. no)									
Ill health	0.779	0.114	***	0.752	0.113	***	0.733	0.113	***
Child social class (ref. Professional)									
Non-manual skilled	0.255	0.255	*	0.243	0.243	*	0.230	0.230	*
Manual skilled	0.524	0.524	***	0.494	0.494	***	0.452	0.076	***
Semi-skilled/unskilled	0.638	0.086	***	0.586	0.084	***	0.524	0.081	***
Adult social class (ref. Professional)									
Non-manual skilled	0.247	0.057	***	0.218	0.056	***	0.210	0.056	***
Manual skilled	0.408	0.060	***	0.366	0.060	***	0.352	0.060	***
Semi-skilled/ unskilled	0.521	0.081	***	0.474	0.079	***	0.457	0.080	***
Marital status (ref. unmarried)									
Married	−0.361	0.041	***	−0.287	0.040	***	−0.279	0.041	***
N	80,086			80,086			80,086		

Note: *** *p*-value < 0.001, ** *p*-value < 0.01, * *p*-value < 0.05.

## Data Availability

The data presented in this study are available on request from the Centre for Longitudinal Studies: https://cls.ucl.ac.uk/. Data are not publicly available due to potential identifiability issues.
